# Modified two-needle technique of air/fluid exchange for in-office treatment of unhealed macular hole

**DOI:** 10.1186/s40942-025-00705-1

**Published:** 2025-07-14

**Authors:** Jun Chen, Shiya Mai, Yayun Wang, Yi Yu, Mo Tian, Xuhui Yu

**Affiliations:** Department of Ophthalmology, The First Affiliated Hospital of Harbin Medical University, No.143, Yiman Road, Harbin City, Heilongjiang Province 150001 China

**Keywords:** Unhealed macular hole, Air-fluid exchange, In-office treatment

## Abstract

**Background:**

Pars plana vitrectomy (PPV) combined with internal limiting membrane (ILM) peeling and sterilized air tamponade is used to treat macular hole(MH). Unsuccessful closure of the macular hole may occur after PPV, some caused by insufficient air tamponade or incorrect position. In-office air-liquid exchange may be an option for these patients.

**Methods:**

A modified two-needle method of air-fluid exchange in office is introduced. A 29-gauge needle is inserted 3.5-mm posterior to the limbus at 6 o’clock. A second 29-gauge needle with a 5mL syringe filled with sterile air is inserted 3.5-mm posterior to the limbus in the superotemporal quadrant or superonasal quadrant. The plunger of the air-filled syringe is pushed while liquid of vitreous cavity flows out of the 6 o’clock needle drop by drop, and the flow rate of the fluid changes with the injection pressure.

**Results:**

The method approximates the conditions of air-fluid exchange in vitrectomy, and the air injection and liquid outflow are balanced by pressure naturally.

**Conclusion:**

The modified two-needle method is an easy, safe, and effective in-office air-fluid exchange for the treatment of unhealed macular holes.

## Introduction

Pars plana vitrectomy (PPV) combined with internal limiting membrane (ILM) peeling and gas tamponade remains the gold standard for macular hole (MH) repair, achieving anatomical closure rates exceeding 90% in most series [1]. Persistent macular holes following initial surgery may require extended tamponade duration, where sterile air emerges as a viable alternative to traditional sulfur hexafluoride (SF6) or perfluoropropane (C3F8) gases [2, 3]. Comparative studies demonstrate equivalent closure rates between air and gas tamponade, with air offering distinct advantages, including rapid resorption (2–5 days vs. 2–6 weeks) and reduced risk of prolonged intraocular pressure(IOP) elevation [4, 5].

While operating room-based air-fluid exchange under sterile conditions remains an option, its technical complexity and logistical constraints have driven the development of in-office procedures. Current in-office techniques include: Single-needle open systems requiring simultaneous infusion and aspiration, dual-port open systems with separate infusion and drainage, the modified dual-needle closed system (MDNCS) employing a 27G drainage needle and 30G air infusion cannula, etc [6–10]. A different method of air-fluid exchange can be accompanied using a modified two-needle system that utilizes a needle for liquid drainage and a second needle with syringe for air injection. The purpose of this article is to describe in detail the modified two-needle technique for air fluid exchange in-office and compare it with other techniques.

## Technique

**Fig. 1:**
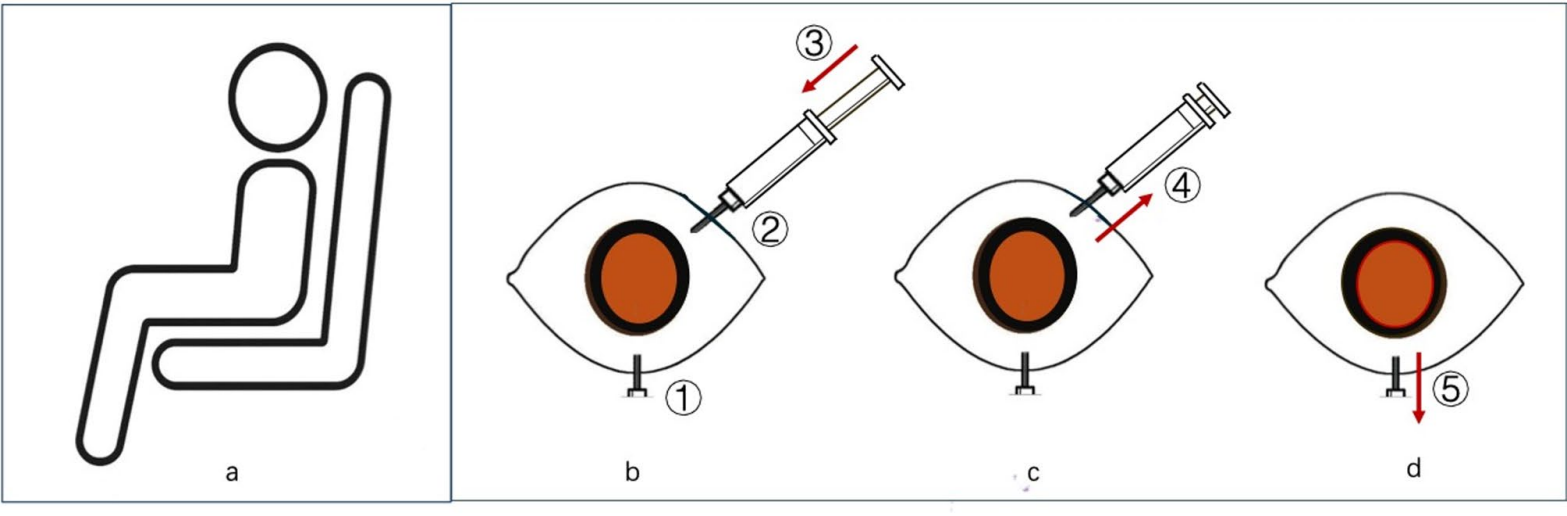
Schematic diagram of the modified two-needle technique of air/fluid exchange. Schematic diagram of modified two-needle technique of air/fluid exchange: patient took the seated position (**a**); first, insert a 29-gauge needle 3.5 mm posterior to the limbus at 6 o ‘clock into the vitreous cavity(①); second, insert a 29-gauge needle with a 5mL sterile air syringe 3.5 mm posterior to the limbus in the supertemporal quadrant or super-nasal quadrant into the vitreous cavity(②); third, push the plunger of the air-filled syringe and the fluid from the vitreous cavity comes out drop by drop from the 6 o’clock needle(③)(**b**); four, pull out the needle with the syringe(④)(**c**); five, pull out the needle at 6 o ‘clock(⑤)(**d**)

Prepare the materials needed for the method, including a 29-gauge needle and a syringe with 5mL of sterile air equipped with a 29-gauge needle. The patient takes a seated position (shown in Fig. 1(a)). After topical anesthesia, the eye is rinsed with 5% povidone iodine solution and the eyelid speculum is placed.

First, a 29-gauge needle without a syringe is inserted 3.5 mm posterior to the limbus at 6 o ‘clock through the pars plana into the vitreous cavity (shown in Fig. 1(b)①). Then, a second 29-gauge needle with a 5mL sterile air syringe is inserted 3.5 mm posterior to the limbus in the supertemporal quadrant or super-nasal quadrant into the vitreous cavity (shown in Fig. 1(b)②). Third, the plunger of the air-filled syri1nge is pushed and the fluid from the vitreous cavity comes out drop by drop from the 6 o’clock needle in a rate of no more than 60 drops per minute (shown in Fig. 1(b)③). The flow rate of the fluid matches the pressure drop of the eye automatically. The procedure is continued until air is observed in the 6 o’clock needle. Subsequently, the needle with the syringe is pulled out first (shown in Fig. 1(c)④), and then the needle at 6 o ‘clock is also pulled out (shown in Fig. 1(d)⑤), at which time the IOP is slightly lower than normal. Vision is then confirmed to be at least hand motion and topical antibiotics is applied to the eyes prior to the patients leaving the clinic. Patients are asked to remain a prone position for 3–5 days.

### Patient characteristics and procedures outcomes

We conducted a retrospective, consecutive case series study that included three patients (three eyes) treated at the Eye Hospital of the First Affiliated Hospital of Harbin Medical University between January 2024 and April 2025. All patients were diagnosed with macular holes(MH) and Gass classification ≥ grade 3. Patients were considered for in-office air-fluid exchange if they have with unsealed hole approximately 1 week after macular hole surgery without other post-operative complications, and spectral domain optical coherence tomography (OCT) (Spectralis OCT; Heidelberg Engineering, Heidelberg, Germany) showed that the diameter of the MH is less than 500 μm. The surgery was performed by an experienced surgeon (XH Yu). Table 1 lists the clinical characteristics and treatment results of the patients in detail.

**Fig. 2:**
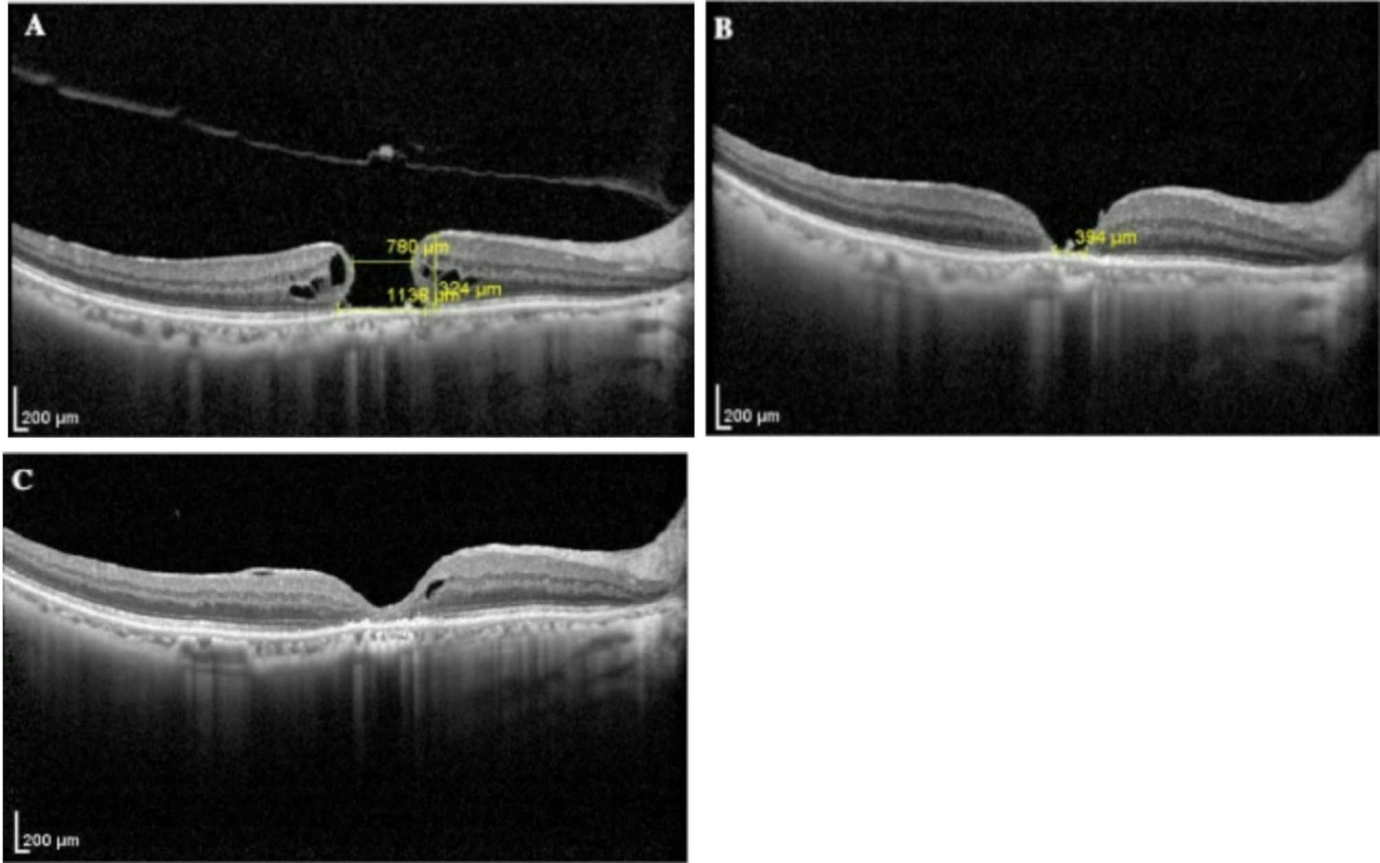
Sequential records of the size of macular holes in patient 1. (**A**) Preoperative assessment: OCT demonstrated a full-thickness macular hole with basal diameter 1,138 μm (3/4PD) and minimum linear diameter 780 μm. (**B**) Postoperative assessment(POD 10): OCT revealed persistent but reduced macular defect prior to air-fluid exchange. (**C**) Final follow-up (3 months post-exchange): OCT confirmed complete anatomical closure with restoration of normal foveal contour

A 76-year-old female (Patient 1) presented to our department with a 12-month history of diminished visual acuity and metamorphopsia in the right eye. Her medical history included hypertension and diabetes mellitus. Initial best-corrected visual acuity (BCVA) in the affected eye was counting fingers(CF). IOP measured 16 mmHg. Slit-lamp biomicroscopy revealed an unremarkable anterior segment apart from lens opacity. Following mydriasis, 90D lens fundoscopy demonstrated a dark red, well-circumscribed macular hole measuring approximately 3/4 disc diameters (PD) (Fig. 2A). The patient underwent 23-gauge PPV with ILM peeling and sterile air tamponade together with cataract phacoemulsification and intraocular len implantation. On postoperative day 10, partial closure was observed with residual hole dimensions of 1/4 PD (Fig. 2B), accompanied by BCVA improvement to 0.3. An in-office air-fluid exchange was subsequently performed. Further reduction in hole size was noted by day 9 post-exchange. Three months postoperatively, BCVA improved to 0.5 with OCT confirming U-shaped macular architectural restoration (Fig. 2C).

**Fig. 3:**
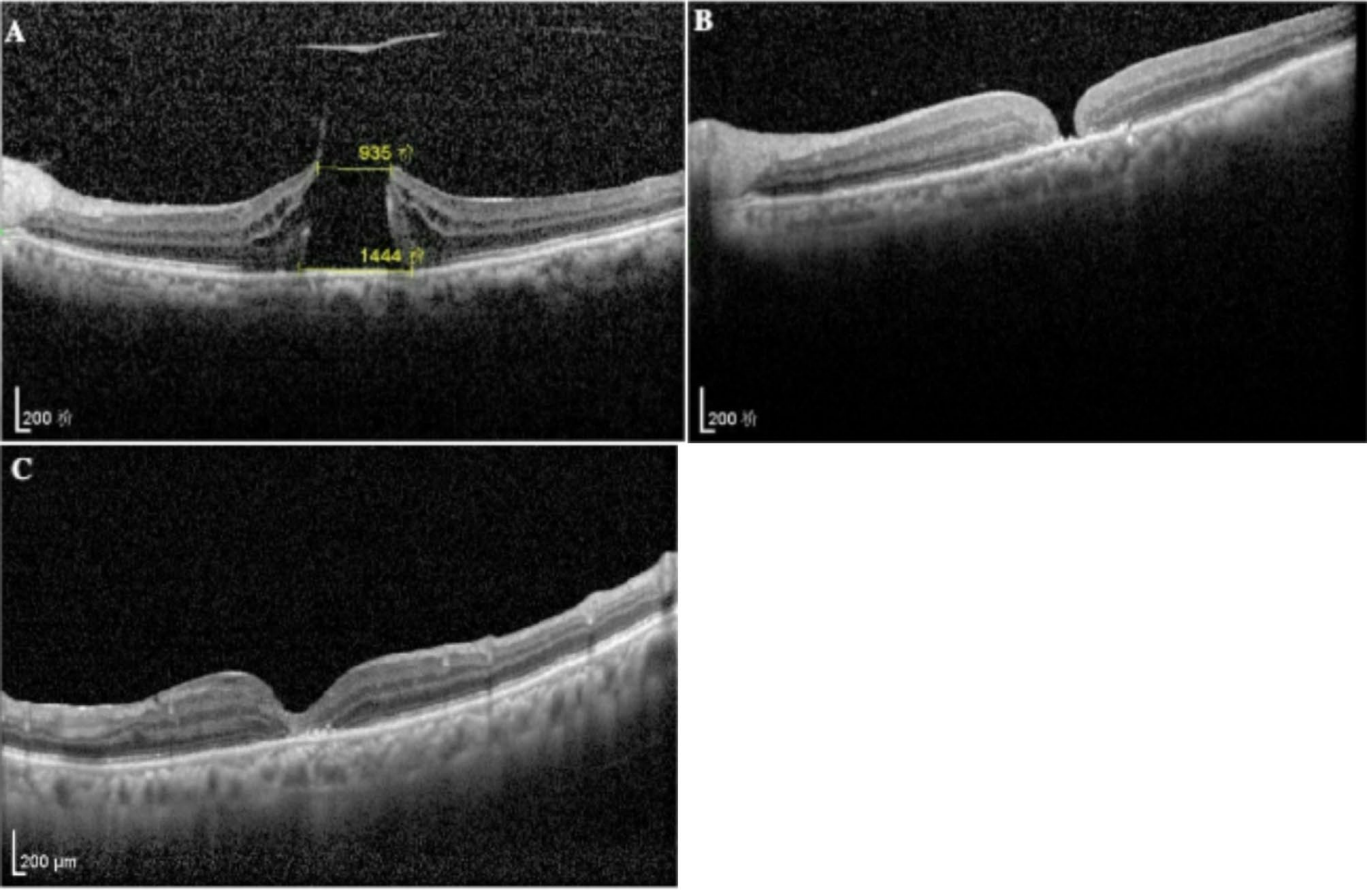
Morphological evolution of macular hole in Patient 2. (**A**) Baseline imaging: OCT revealed an irregular full-thickness macular hole with basal diamete 1444 μm(1PD) and associated ERM. (**B**) Post-vitrectomy status (POD 7): OCT documented an unclosed macular hole preceding in-office air-fluid exchange. (**C**) Post-intervention outcome (3 months): complete anatomical closure with physiological foveal restoration was observed on OCT

Patient 2, a 68-year-old woman with a 10-year history of hypertension and 4-year history of type 2 diabetes mellitus, presented to our clinic with a 1-year history of left-eye visual decline. She was diagnosed with a macular hole and epiretinal membrane(ERM) with diabetic retinopathy. The BCVA of the left eye was 0.04. IOP was 15 mmHg. Anterior segment examination was unremarkable. Dilated fundus examination revealed venous tortuosity, microaneurysms, and hemorrhages. A dark red, irregularly bordered full-thickness macular hole measuring 1 PD was visualized within the posterior pole, exhibiting aberrant reflectivity (Fig. 3A). Standard PPV with ILM peeling was performed. One week postoperatively, persistent macular defect (1/4 PD) was observed (Fig. 3B) with no significant improvement in BCVA.In-office air-fluid exchange was performed. One week late, OCT demonstrated complete U-shaped closure confirmed by OCT (Fig. 3C). At the 6-month follow-up, BCVA had increased to 0.2 without recurrence.

**Fig. 4:**
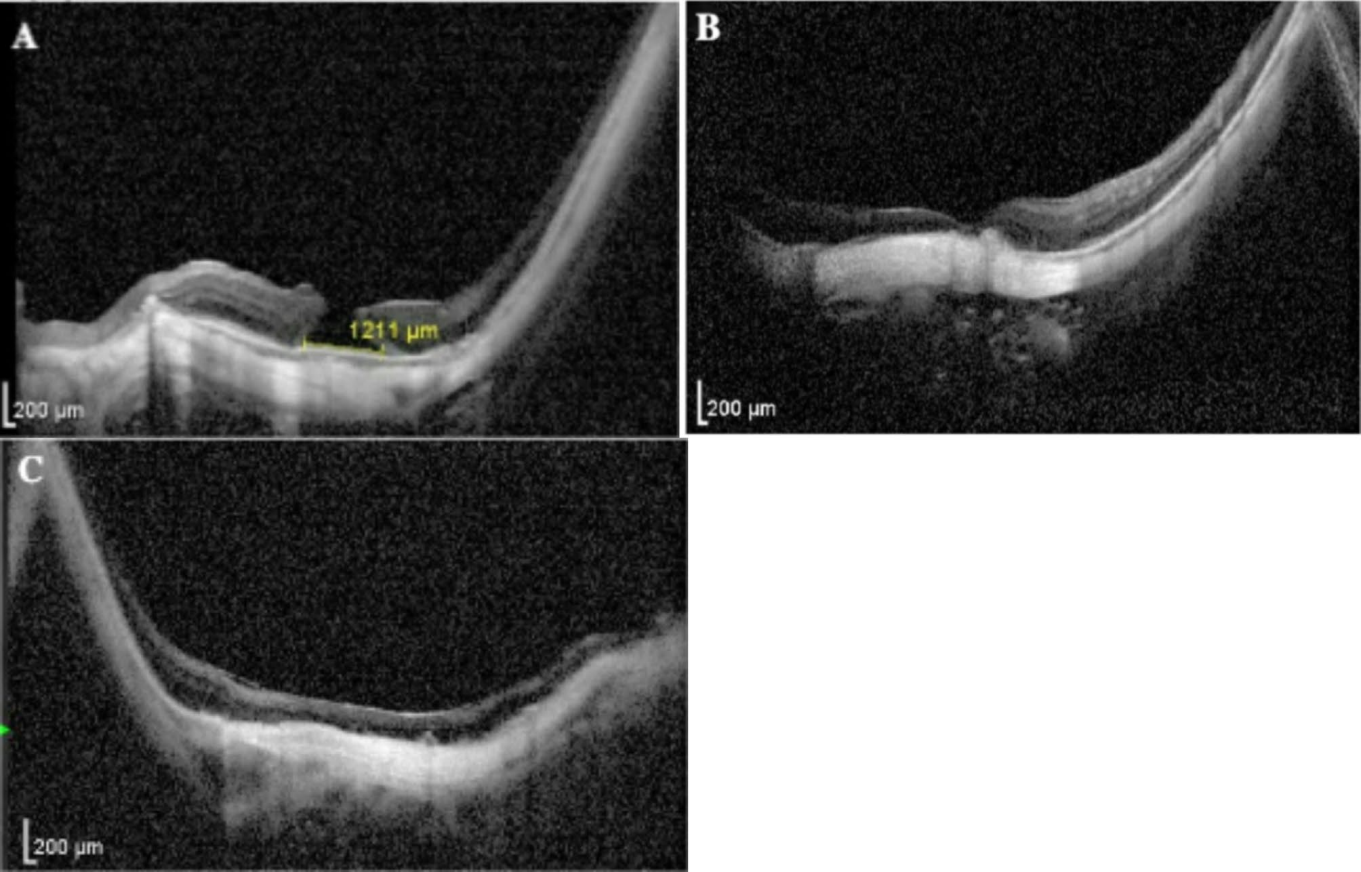
Sequential changes in high-myopia macular hole (Patient 3). (**A**) Preoperative evaluation: OCT demonstrated a 1211 μm (3/4 PD) macular hole within characteristic myopic maculopathy, featuring leopard-spot pigmentary changes and peripapillary atrophy. (**B**) Postoperative phase (POD 7): a persistent but reduced macular hole visualized before air-fluid exchange. (**C**) Outcome (4 months post-exchange): OCT confirmed U-shaped foveal reconfiguration

A high myopia patient (Patient 3) presented with progressive left-eye visual deterioration over a 24-month period. The BCVA was 0.02. IOP was 13 mmHg. Dilated fundus examination and imaging including OCT and fundus photography identified characteristic high-myopia features including leopard-spot pigmentary changes, peripapillary atrophy, *Fuchs* spots, and a 3/4 PD macular hole (Fig. 4A). Post-vitrectomy assessment at 1 week showed a reduced but persistent hole (Fig. 4B), with BCVA improvement to 0.15. The in-office air-fluid exchange technique was performed, achieving successful closure. Four-month follow-up OCT demonstrated U-shaped macular reconfiguration (Fig. 4C) with corresponding BCVA recovery to 0.2.

**Table 1:** Baseline characteristics of each patient and clinical results of modified two-needle technique of air/fluid exchange for in-office

	Patient 1	Patient 2	Patient 3
Age(y)	76	70	71
Sex	Female	Female	Female
Eye	Right	Left	Left
Size of MH before vitrectomy (µm)	1138	1444	1211
Stage of Macular Hole	4	4	4
Macular Hole Duration (mo)	12	12	24
Initial BCVA	CF	0.04	0.02
Size of MH one weeks after vitrectomy (µm)	394		
MH closure time (days)	14	7	
Final Visual Acuity	0.5	0.2	0.2
Type of OCT Image	U	U	U
Complications	None	None	None

## Discussion

The modified two-needle in-office air-fluid exchange technique also demonstrates promising clinical efficacy in managing select cases of persistent macular holes post-vitrectomy, as evidenced by its successful application in our initial series of 10 eyes.No recurrences were observed during the follow-up period, which included a maximum duration of 6 months. BCVA improved in all cases. The final macular morphology was either U-shaped (normal foveal contour) or V-shaped (steep foveal contour) on OCT images. At the current follow-up examination, none of these patients showed complications. None of the patients required follow-up surgery.

The previously conventional in-office open system had shown good results in treating unhealed MH after surgery. The modified two-needle technique offers three principal advantages over conventional methods.

First, the enhanced operative efficiency stems from multiple innovations: (1) Maintaining patients in an upright seated position eliminates the logistical challenges and patient discomfort associated with prone/supine positioning documented in prior studies [9, 11]; (2) The minimalist instrumentation requirement - utilizing only two 29-gauge needles and a single 5 mL air-filled syringe - significantly reduces setup complexity; (3) The standardized procedural sequence (initial inferior 6-o’clock drainage needle placement followed by superior quadrant infusion needle insertion) ensures reproducible execution. Controlled air injection through the superior port creates a pressure gradient that induces passive fluid egress via the inferior needle, thereby maintaining dynamic IOP equilibrium throughout the exchange process.

Second, the technique achieves remarkable time efficiency through its self-regulating fluid dynamics. Based on our calculated displacement rate of 0.05 mL per droplet (estimated) at 1 droplet/second, complete 5 mL fluid exchange can be reliably accomplished within ≤ 2 min. Procedural termination requires only sequential needle withdrawal - first the superior infusion needle followed by the inferior drainage port - without necessitating additional closure steps or postoperative interventions.

Third, this operation process is based on the effect of fluid mechanics to maintain the intraocular pressure stable at a level slightly lower than the initial intraocular pressure, but still within the normal intraocular pressure range. In the modified double-needle technique, after the needle at the 6 o ‘clock position is inserted into the vitreous cavity, a small amount of intraocular fluid moves into the needle under the action of atmospheric pressure, and even one drop may drip, causing the IOP to be slightly lower than the initial intraocular pressure. When the upper needle is then inserted and the air injection begins, the change in IOP caused by the air injection prompts the intraocular fluid to flow out through the lower needle. The speed of fluid outflow varies according to the injection pressure, achieving dynamic balance regulation of intraocular pressure, thereby keeping the IOP slightly lower than the normal level of the initial intraocular pressure throughout the process. At the end of the injection, the upper needle is removed first, and then the drainage needle at the 6:00 o ‘clock position is removed - ultimately, the IOP remains at a safe level slightly below the initial intraocular pressure. Continuous IOP self-regulation prevents the dangerous pressure fluctuations characteristic of single-needle open systems, which have been associated with 18–35 mmHg instantaneous pressure variations in previous reports [9]. By decoupling the infusion and drainage functions into separate ports, the technique eliminates the operator coordination errors inherent in “simultaneous syringe” approaches that predispose to iatrogenic IOP spikes [6]. Furthermore, the single-operator feasibility removes assistant-dependent variables, reducing staffing requirements while improving procedural consistency.

One significant limitation of this technique is that a sterile collection device located below the lower needle (at the 6 o’clock direction) should be used to prevent iatrogenic contamination. If the patient holds the container during air-fluid exchange, the patient’s anxiety about the progress of eye operations will also be partially alleviated.

## Conclusion

In conclusion, the modified two-needle technique is a simple, safe and convenient air-fluid exchange procedure for patients who are suitable for this treatment.

## Data Availability

No datasets were generated or analysed during the current study.

## Abbreviations

PPV: Pars plana vitrectomy
ILM: Internal limiting membrane
MH: Macular hole
IOP: Intraocular pressure
MDNCS: Modified dual-needle closed system
OCT: Optical coherence tomography
mm: Millimeters
mL: Milliliters
